# Perceived dilemma between protective measures and social isolation in nursing homes during the COVID-19 pandemic: a mixed methods study among Swiss nursing home directors

**DOI:** 10.3389/fpubh.2024.1292379

**Published:** 2024-03-11

**Authors:** Stephanie Greindl, Stefania Di Gangi, Andreas Plate, Oliver Senn, Stefan Neuner-Jehle

**Affiliations:** Institute of Primary Care, University of Zurich and University Hospital of Zurich, Zurich, Switzerland

**Keywords:** COVID-19 pandemic, protective measures, social isolation, nursing homes, nursing home residents, ethical dilemma, embedded mixed-method study

## Abstract

**Background:**

Coronavirus pandemic (COVID-19) particularly affected older adults, with the highest risks for nursing home residents. Stringent governmental protective measures for nursing homes unintendedly led to social isolation of residents. Nursing home directors (NDs) found themselves in a dilemma between implementing protective measures and preventing the social isolation of nursing home residents.

**Objectives:**

The objectives of this study were to describe protective measures implemented, to investigate NDs’ perception of social isolation and its burden for nursing home residents due to these measures, and to explore experiences of NDs in the context of the dilemma.

**Methods:**

Cross-sectional embedded mixed-method study carried out by an online survey between April 27 and June 09, 2022, among NDs in the German-speaking part of Switzerland. The survey consisted of 84 closed-ended and nine open-ended questions. Quantitative findings were analyzed with descriptive statistics and qualitative data were evaluated using content analysis.

**Results:**

The survey was completed by 398 NDs (62.8% female, mean age 55 [48–58] years) out of 1′044 NDs invited.

NDs were highly aware of the dilemma. The measures perceived as the most troublesome were restrictions to leave rooms, wards or the home, restrictions for visitors, and reduced group activities. NDs and their teams developed a variety of strategies to cope with the dilemma, but were burdened themselves by the dilemma.

**Conclusion:**

As NDs were burdened themselves by the responsibility of how to deal best with the dilemma between protective measures and social isolation, supportive strategies for NDs are needed.

## Introduction

In March 2020, the World Health Organization (WHO) declared the coronavirus disease 2019 (COVID-19) outbreak a global pandemic (1). Older adults were particularly affected (2, 3) and had increased risks for hospitalization (2, 4) and death (5). Nursing home residents were at particular high risk as they combined key risk factors for severe or fatal COVID-19 courses, such as advanced age and co-morbidity. Accordingly, the mortality rates were highest in nursing homes, especially at the beginning of the pandemic (6).

After the first confirmed COVID-19 case in Switzerland on February 25, 2020 and the subsequent increase in infection numbers (7), restrictive measures were gradually introduced to protect the overall population and high-risk populations like nursing home residents (8, 9). As an example the number of contacts (communal dining, group activities, etc.) and social activities were reduced (10). Additionally, contacts with all kinds of healthcare providers were kept to a minimum and suspected cases were confined to their rooms (11). On the international level, the majority of nursing facilities were affected similarly by the thread of the COVID-19 pandemic and the necessity to protect their residents (12).

In Switzerland, as in many other countries, nursing home directors (NDs) are responsible for the governance of structures, procedures and outcomes of healthcare in their nursing home facilities, in order to guarantee the quality of care for residents (13). Nursing home directors (ND), in their function to manage the nursing staff and overall nursing operations of their healthcare facility, were in a difficult situation and faced a dilemma (14): On one hand, they were responsible for the protection of nursing home residents from infection with a potentially life-threatening disease. On the other hand, they perceived that the protective measures negatively affected their nursing home residents (15). In addition, it was more difficult for NDs to cope with this dilemma due to different and even contradicting recommendations by regional and national authorities. Of note, Swiss healthcare governance is organized in a combined manner, both regionally and federally.

To our knowledge, it is unknown how NDs in Switzerland have experienced this dilemma and how they have dealt with it. The knowledge about their experience and coping strategies would allow developing strategies for NDs and their teams, in favor for a better preparedness for future pandemics. Thus, the aim of this study was to describe: (i) the implemented protective measures reported by NDs, (ii) the NDs’ perception of social isolation among nursing home residents due to the implemented protective measures, (iii) the NDs’ perception of the burden among nursing home residents due to this social isolation, and (iv) to explore perceptions and experiences among NDs in the context of the dilemma.

## Methods

### Study design/research design

Cross-sectional embedded mixed-methods survey study. The survey was implemented as a self-administered online survey using the survey software REDCap (Research Electronic Data Capture) (16). The authors decided to perform an embedded mixed-method study in order to obtain both quantitative and qualitative results regarding the dilemma (17).

We developed the item set of our survey instrument following the rationale of our research questions. Taking into account the explorative design of our study, there was no option to base our instrument on existing theoretical constructs nor on validated item sets.

### Participants/study population

All nursing homes (*n* = 1,044) providing care for their residents either continuously (long-term care) or on a 24-h stand-by care base in the German-speaking part of Switzerland were eligible for participation. Nursing homes were identified using data provided by the Swiss Federal Statistical Office. All nursing homes, which were listed by the Swiss Federal Statistical Office at that time and fulfilled the inclusion criteria were invited to participate in the study. Therefore, the inclusion criteria were as follows: (1) Nursing Home in the German-speaking part of Switzerland, (2) Nursing Home where older adults in need of care are housed, (3) Nursing home where older adults are provided with 24-hours care and nursing services and, (4) Nursing home with long-term care (LTC) services. Nursing homes in which residents do not stay overnight were excluded. NDs were invited to participate in the survey study by email. The survey was open from April 27 to June 09, 2022. We sent two reminders within four weeks.

### Survey instrument

The survey was piloted by a random sample of 30 NDs providing feedback on the comprehensibility of the questions, user-friendliness, technical functionality, and the content of the survey.

The final survey included seven chapters corresponding to the following themes: (i) physical distancing, (ii) visitors’ restrictions, (iii) restrictions to physicians and other healthcare providers’ contacts, (iv) restrictions to group activities, (v) NDs experiences in handling the dilemma, (vi) ND’s burden during the pandemic, and (vii) basic data of the participating NDs and the nursing homes. The items related to the entire period of the pandemic were stated at the beginning of the questionnaire. The questionnaire included closed-ended (multiple-choice and Likert style) and open-ended questions. In addition, all questions offered a response option of “Do not know,” “Not applicable,” or “I cannot evaluate.” Participants were able to check their answers at any time during the survey access and edit them if necessary. The total survey consisted of 93 questions, in German language, of which 84 were quantitative and nine were qualitative. An English translation and further information regarding the survey are provided in the Supplementary material 1.

### Data analysis

We defined our response rate as the number of participants who answered at least one item, divided by the number of all invited participants (denominator). Due to the explorative nature of this survey study, we analyzed all available answers. The exact numbers of analyzed answers for each item is given in the results part.

#### Quantitative analysis

We performed statistical analysis using the statistical package R version 4.1.0 (18). We used descriptive statistics to characterize the nursing homes and the NDs. Data was checked for plausibility before analysis, and we excluded answers outside the valid value range from analysis. In the case of missing data in a given item, the nursing home was excluded from analysis for that item only.

#### Qualitative analysis

Each of the nine open-ended questions represented a pre-defined dimension of interest. Additionally, we pre-defined another dimension (for residents with dementia and cognitive impairment), without a specific question. Within each of these dimensions we identified themes and subthemes using the method of qualitative text analysis in a systematic and rule-based way described by Kuckartz (19). We used the triangulation method to ensure intersubjectivity, as follows: Four researchers coded the transcripts independently and met regularly to reconcile coding differences and to identify themes and subthemes. The research team discussed and determined the codes in five face-to-face meetings, and went through all of the analysis steps described above until consensus was reached. Data organization and analysis were performed using the software MAXQDA (20).

This study was reported according to the Checklist for Reporting Results of Internet E-Surveys (CHERRIES) (Supplementary material 2) (21) and the COREQ Checklist for Reporting Qualitative Results (22) (Supplementary material 3).

### Ethics

Due to the nature of the study, no ethics approval was necessary and the local Ethics Committee of the Canton of Zurich confirmed that the study did not fall under the scope of the national Human Research Act (BASEC-Nr. Req-2022-00153). Participation in the survey was voluntary and all participants accepted a general informed consent informing them about the purpose and aims of the study. All participants agreed to the publication of their anonymized data. The study was conducted in accordance with the Declaration of Helsinki (23).

## Results

The survey was sent to 1,044 NDs and *n* = 398 filled out the questionnaire, resulting in a response rate of 38.1%. Baseline sociodemographic characteristics of the NDs and basic information about the analyzed nursing homes are shown in Supplementary material 4.

The most common reported *protective measures* were visitors regulations according to national regulations (*n* = 342, 97.7%), limitation in visitor numbers (per visit) (*n* = 335, 95.7%), and a visit ban allowing exceptions in special circumstances (emergencies or palliative situations) (*n* = 306, 87.2%). The less common reported protective measures were an absolute visit ban without any exceptions (*n* = 27, 7.7%), staggered mealtimes (*n* = 94, 23.6%), and a ban to leave one’s own private room (*n* = 183, 46.1%) (Table 1).

**Table 1:** Implementation of protective measures in the nursing homes.

Have you introduced the following protective measures at any time in your institution?	*n*	Protective measure implemented		
		Yes (*n*)	No (*n*)	Do not know (*n*)
Physical distancing				
Distancing rules	397	336 (84.6)	59 (14.9)	2 (0.5)
Staggered mealtimes	398	94 (23.6)	300 (75.4)	4 (1.0)
Restrictions for using common rooms	398	242 (60.8)	154 (38.7)	2 (0.5)
Ban to leave private rooms	397	183 (46.1)	211 (53.1)	3 (0.8)
Ban to leave the ward	395	223 (56.5)	169 (42.8)	3 (0.8)
Ban to leave the nursing home	397	263 (66.2)	132 (33.2)	2 (0.5)
Visitors’ restrictions				
Limitation in visitors’ number (per visit)	350	335 (95.7)	14 (4.0)	1 (0.3)
Limitation in visitors’ number (per day/week)	350	248 (70.9)	100 (28.6)	2 (0.6)
Visits by closest family members/friends only	351	212 (60.4)	133 (37.9)	6 (1.7)
Visits in visitors’ rooms only	351	261 (74.4)	88 (25.1)	2 (0.6)
Outdoor visits only	351	252 (71.8)	93 (26.5)	6 (1.7)
Visitors’ rules according to national regulations*	350	342 (97.7)	6 (1.7)	2 (0.6)
Visit ban, exceptions only in special circumstances (emergencies or palliative situation)	351	306 (87.2)	44 (12.5)	1 (0.3)
Visit ban, no exceptions	350	27 (7.7)	320 (91.4)	3 (0.9)

*Regulations were: (1) vaccinated or recovered or tested (negative PCR test) or (2) vaccinated or recovered, or (3) vaccinated or recovered or tested (negative antigen rapid test).

*Changes in the provision of care* due to the implemented protective measures are described in Table 2. Core services such as physician home visits (*n* = 235, 68.7%), assistance in personal care & hygiene (*n* = 295, 86.3%), assistance in mobility (*n* = 249, 72.8%), psychological care by nurses (*n* = 236, 69.0%) and spiritual end-of-life care (*n* = 226, 66.1%) were perceived as unchanged by NDs. A majority of NDs (*n* = 268, 81.9%) reported that there was no or only a moderate negative impact of the pandemic on the quality of care provided by physicians, with similar results for non-physician care (*n* = 257, 78.6%).

**Table 2:** Change in the provision of care or activity by physicians, other healthcare providers and nursing home staff.

How have the following type of care or activities (considering contacts between provider and resident) been changed?	*n*	Provision of care or activity			
		Unchanged (*n*)	Limited (*n*)	Not provided any more (*n*)	Do not know (*n*)
Physician visits (in the practice)	342	171 (50.0)	132 (38.6)	27 (7.9)	12 (3.5)
Physician visits in the nursing home	342	235 (68.7)	96 (28.1)	7 (2.0)	4 (1.2)
Physiotherapy	342	123 (36.0)	171 (50.0)	47 (13.7)	1 (0.3)
Logopedics	342	105 (30.7)	107 (31.3)	59 (17.3)	71 (20.8)
Assistance in personal care and hygiene	342	295 (86.3)	46 (13.5)	0 (0.0)	1 (0.3)
Assistance in mobility	342	249 (72.8)	92 (26.9)	0 (0.0)	1 (0.3)
Psychological care by nurses	342	236 (69.0)	101 (29.5)	0 (0.0)	5 (1.5)
Pastoral care	342	114 (33.3)	168 (49.1)	58 (17.0)	2 (0.6)
Spiritual end-of-life care	342	226 (66.1)	95 (27.8)	11 (3.2)	10 (2.9)
Frequency of group activities	320	69 (21.6)	196 (61.3)	54 (16.9)	1 (0.3)
Limitation of participants’ number for group activities	320	71 (22.2)	203 (63.4)	45 (14.1)	1 (0.3)

**Figure 1:**
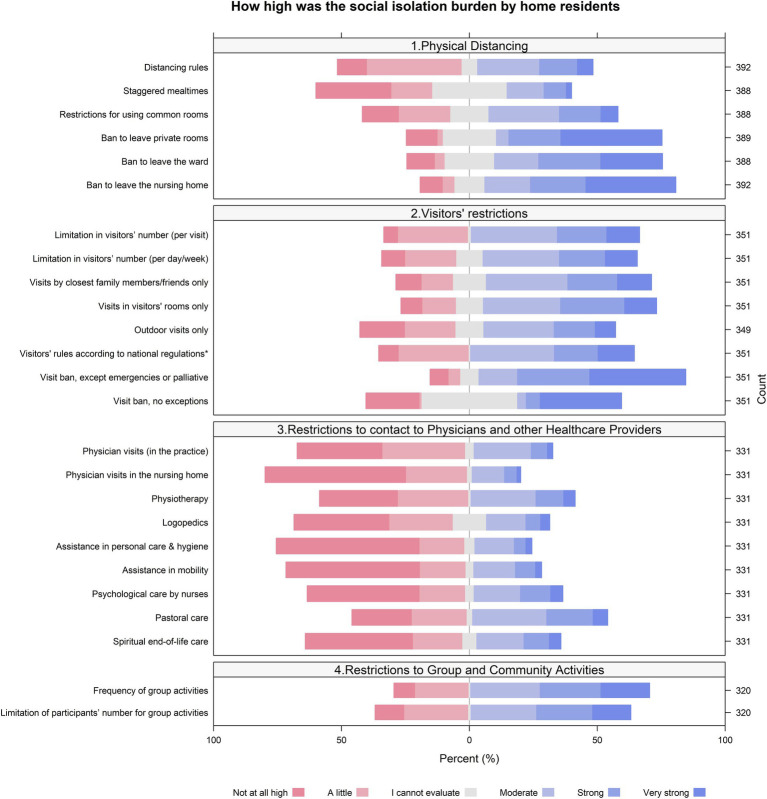
Perceived (by NDs) social isolation among nursing home residents due to protective measures. (Answers to the item “To what extent has the protective measure led to social isolation among nursing home residents?”).

**Figure 2:**
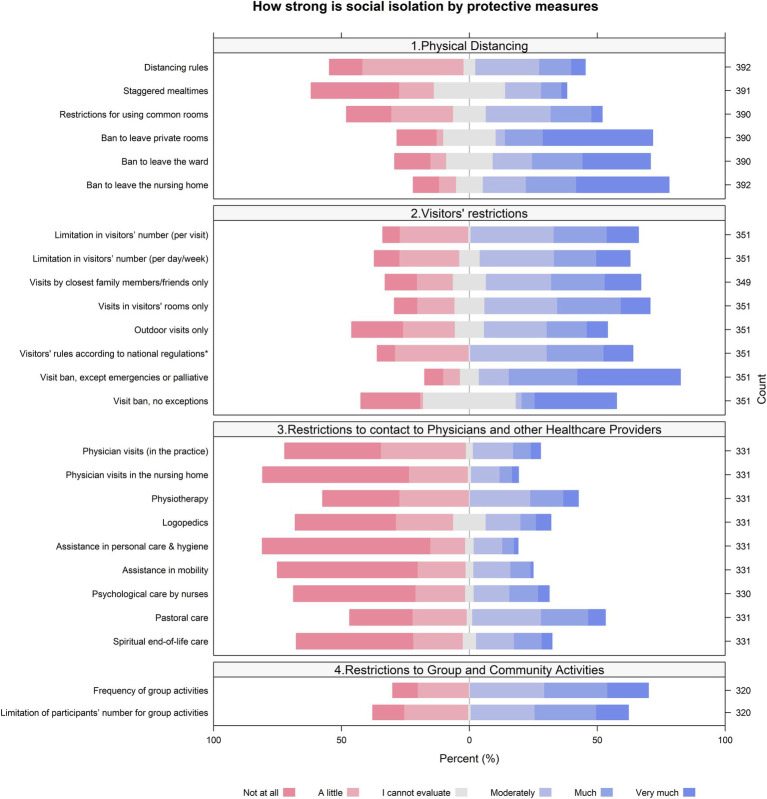
Perceived (by NDs) burden of the social isolation among nursing home residents. (Answers to the item “What do you think: How high was the burden of this social isolation for the home’s residents?”).

We found that both the social isolation among nursing home residents, due to the protective measures, Figure 1, and the burden of this social isolation as perceived by the NDs, Figure 2, were highest for bans to leave private rooms, wards or the home, for visitor restrictions and for restricted group and community activities. On the other hand, protective measures leading to a restriction of contacts with health care providers contributed less to social isolation and burden of social isolation.

### Qualitative survey results

In this chapter we present the main qualitative findings for all dimensions which were pre-defined by the corresponding open-text survey question (1–9). Additionally, we added one dimension for the findings related to residents with dementia and cognitive impairment. In each dimension section, we present the findings as themes, subthemes and corresponding quotes, according to our analysis. An overview table of dimensions, themes, subthemes and citations is provided in Supplementary material 5.

#### Negative impact on medical service quality

Three themes were identified: limitation of availability of physicians, overload, and inter-professional collaboration.

The NDs experienced that occasionally there was no or limited availability of physicians. Sub-themes included self-protection from infection, accessibility, and medical prioritization. One respondent stated:

*“Physicians are visiting patients in severe cases only” (Respondent 328, Nursing Home Manager, 62 years, Line 66).*

In addition, physicians’ high workload and shortage of time were identified as another reason for reduced medical service quality, with one participant concluding:

*“General practitioners were overwhelmed by the situation and had fewer resources for the nursing homes” (Respondent 266, Nursing Home Manager, 47 years, Line 55).*

As a next point, the shortage of healthcare workers was specified. Respondent 37 noted:

*“The lack of professionals, such as physicians, pharmacists, physiotherapists,* etc. *is fundamentally a problem” (Respondent 37, Nursing Home CEO, 54 years, Line 11).*

As a last theme, the inter-professional collaboration between the nursing home care team(s) and physicians was perceived as more difficult and poorer and medical tasks were more often shifted to the nursing home care team(s).

#### Negative Impact on non-medical service quality

The negative impact on non-medical service quality themes included the two themes: high workload, and the reduction of non-nursing therapy services.

The NDs reported a reduction or omission of non-medical healthcare, e.g., physiotherapy, activation therapy or spiritual care, due to the high workload. The reasons for this were a shortage of time, with one respondent stating:

*“Organizational issues, communication, documentation and protective measures required a lot of time, so there was less time for the care of residents” (Respondent 9, Nursing Home Manager, 50 years, Line 105).*

Furthermore, the NDs identified the fundamental shortage of healthcare professionals as the reason for the negative impact on the non-medical service quality.

#### In-house measures to cope with the dilemma

The NDs reported the following four measures that they had implemented to reduce the dilemma: activation offerings, internal and external communication, liberal implementation of protection measures, and recruitment of personnel. NDs stated that they started innovative offers, e.g., small group or individual activities, or new offers, as shown by the following responses:

*“There were more individual visits by spiritual carers, skype for residents and next of kin, setup of a visitors’ room” (Respondent 32, Nursing Home Manager, 59 years, Line 231).*

*“Concerts to join on the balcony, a visitors’ tent, tablets for using zoom, volunteers for visiting residents individually, taking a walk with them, having a phone or skype call with them, and writing a letter to them. School classes were asked for support by writing letters or giving a drawing as a present” (Respondent 41, Head of Nursing Service, 46 years, Line 237).*

Internal and external communication with residents was intensified. Protective measures were handled liberally. One respondent noted:

*“We tried to find an optimal balance between protective measures and ‘let live’, in agreement with residents and family members” (Respondent 167, Nursing Home Manager, 53 years, Line 299).*

Personnel (nursing and non-specialist staff) was increased. New tasks were taken over by other employees and *“Every employee tried to substitute a part of the lacking offers” (Respondent 287, Nursing Home Manager, 57 years, Line 363).*

#### Support for coping with the dilemma

NDs indicated the following five themes of support for coping: institutes and associations, team, no external support, technical innovation, and no dilemma perceived. The NDs perceived support for coping with the dilemma from official health authorities, professional associations, the care team (i.e., employees, volunteers, spiritual carers, General Practitioners (GPs), family members), the family members, and technical innovations such as social media (e.g., skype, video calls, etc.). As mentioned in one response:

*“There was a strong team spirit and the confidence to manage, together in the multidisciplinary team” (Respondent 219, not specified, not specified, Line 613).*

However, a few NDs perceived no external support or no dilemma at all.

#### Barriers toward coping with the dilemma

The NDs perceived some barriers towards coping with the dilemma. They indicated the following six themes: public authorities and institutions, family members, media, shortage of resources, uncertainty about pandemic progression, and no barrier. There was also a complaint of

*“changing and confusing regulations by the authorities” (Respondent 405, Nursing Home CEO, 58 years, Line 987).*

The NDs stated the misunderstandings of the family members very frequently, with one claiming

*“a poor understanding (of measures) among family members” (Respondent 286, Head of Nursing Service, 58 years, Line 933).*

Others blamed the media for its

“*one-sided reporting” (Respondent 219, Line 899)* of the situation “*not knowing how long and how intensely the pandemic will develop” (Respondent 219, not specified, not specified, Line 899)*.

#### Lessons learned

NDs would adapt concepts, would allow for more autonomy for themselves, or would improve communication. For the adaption of the concept, NDs pointed out the following subthemes: prevent isolation, stock materials, involve family members in decisions, implement escalation levels, and increase key personnel.

Many NDs would allow for more autonomy for themselves, as supported by Respondent 78, who answered:

*“I would hand over more responsibility to the residents and accept their decisions” (Respondent 78, Ward Manager, 50 years, Line 1,109).*

Other NDs would indicate it was important

“*to better communicate with employees, family members (using e-mail) and to better collaborate with authorities*” *(Respondent 363, Line 1,249)* and *“to establish regular consultations for family members” (Respondent 363, Head of Nursing Service, 56 years, Line 1,249).*

#### Potentially positive impact of the pandemic

NDs identified as positive impacts of the COVID-19 pandemic the better preparedness for a next pandemic and the perceived thankfulness of the family members, the team spirit, the solidarity and collaboration between departments. Furthermore, they described that solidarity also came from residents, who were more grateful for the care service. For themselves, NDs identified a higher self-confidence to cope with conditions of crisis, improved digital communication skills (e.g., in the communication with residents and for meetings) and a better understanding and implementation of hygiene measures as positive impacts.

#### Wishes for support for future pandemics

When asked for their wish for support during future pandemics, respondents answered using the following six main themes: media, communication with third parties, resources, autonomy, psychological support, and end-of-life care.

Similar to the findings in dimension 6, a wish for

*“less regulations and more responsibility for the nursing homes and the residents, in accordance with the family members” (Respondent 471*, *Nursing Home Manager, 68 years, Line 1,673)*

Was mentioned. Several NDs would wish for (more) psychological support for themselves. Finally,

“*the topic of dying must be shifted to the society. It has to be possible to openly discuss this with residents and family members” (Respondent 80, Head of Nursing Service, 48 years, Line 1,641).*

#### Further comments related to the dilemma

NDs made further comments related to the impact of the pandemic on residents (e.g., an increase in depressive disorders), family members, care teams, and NDs, indicating

*“There was a huge burden for the care team, physically and psychologically; with no support measures. Many of them quit their jobs after the pandemic” (Respondent 459, Head of Nursing Service, 55 years, Line 2014).*

One respondent stated,

*“The dilemma between protection and autonomy of residents brought me to my limits, and I am still tired and exhausted. The responsibility was enormous” (Respondent 309, Nursing Home Manager, 60 years, Line 1966).*

#### Residents with dementia and cognitive impairment

In this dimension, four themes were identified: communication and understanding, untargeted stimuli, perception, and exceptions from rules and regulations for people with dementia and cognitive impairment.

Communication and understanding of residents were hindered by wearing face masks and reduced capacity of comprehension.

*“Wearing face masks hindered communication and caring substantially, especially for people with cognitive impairment” (Respondent 536, Nursing Home Manager, 54 years, Line 207).*

The decrease of untargeted stimuli resulted in less agitation among the residents.

*“There was less agitation on dementia wards due to less stimuli from outside.” (Respondent 470, Nursing Home Manager, 61 years, Line 1,561).*

The residents did not perceive the protective measures as a restriction.

*“The residents were very relaxed. They did not even perceive the restrictions”(Respondent 472, Head of Nursing Service, Line 736).*

Some authorities allowed less stringent implementation of rules and regulations for residents with dementia and cognitive impairment.

*“The regional authority provided the option that they were allowed having exceptional regulations for people with dementia” (Respondent 95, Nursing Home Manager, 45 years, Line 548).*

## Discussion

### Context and study rationale

During the first part of the pandemic, nursing homes were confronted with the dilemma between the strict implementation of protective measures demanded by national authorities on the one hand, and the resulting social isolation among nursing home residents on the other hand. In our cross-sectional mixed-methods survey study, we explored the perceptions of NDs regarding this dilemma and its consequences for nursing home residents, including factors which hindered or facilitated the coping with this dilemma, and the lessons they learned from this experience.

### Main findings and comparison to existing literature

The perception of NDs of how strong a protective measure led to social isolation was in parallel to their perception of how much this social isolation led to a burden of residents, for the majority of protective measures.

In the perception of NDs, some protective measures were highly troublesome in terms of social isolation and its burden: for example restrictions to leave private rooms, wards or the entire home; restrictions for visitors; reduced group and community activities. In contrast, the restrictions of contacts with health care providers seem to have contributed to social isolation and its burden to a minor degree. The reason for this remains unclear. One may speculate that it was the result of successful coping strategies. For example, NDs’ answered to the question *“What in-house measures were developed to cope with the dilemma?”* that personnel was increased and took over new tasks in order to substitute a part of the lacking offers from other professionals. Another strategy mentioned was technical support by social media, for example by skype or video calls, which may have substituted for visits by professionals partially.

This is in line with the results of other studies investigating the burden of isolation by protective measures and how care teams, residents and families were coping with it (24–26). For example, as reported by the NDs in our study, digital technologies including social media and telemedicine techniques can efficiently support care and fight loneliness of nursing home residents (27–30).

Another finding how NDs and their teams were coping with the dilemma was the architectural adaption, for example cabins for visitors, which allowed them to bypass the strictness of isolation in favor of overcoming the isolation of the residents. This balance is not an easy task, but some experience exists (31).

As nursing home residents frequently suffer from dementia, this important subpopulation deserves special attention. As a highly vulnerable group, with an even higher morbidity and mortality during the COVID-19 pandemic (32), they are exceptionally prone to suffer from a negative impact of social isolation as well (24). Suárez-González et al. stated that *“COVID-19 protection measures have damaged the cognitive and mental health of people with dementia across the world.”* In our study, NDs stated that the communication between care teams and residents with dementia and cognitive impairment was challenging due to wearing face masks and the impaired comprehension. Interestingly, the NDs highlighted some positive aspects of the pandemic for these residents: For example, they often were relaxed due to less untargeted stimuli and they did not perceive protective measures as a restriction for them at all. This is perfectly in line with the findings recently reported by Knippenberg et al. (33).

### NDs’ coping with the dilemma

Our results show that a majority of NDs was highly aware of the ethical dilemma between protective measures and social isolation. They provided a detailed insight into the many ways in which NDs and the nursing home teams were coping with the dilemma. They developed a huge variety of different coping strategies in order to overcome the negative effects of protective measures on social isolation, without violating the protection goal of stopping virus transmission. For successfully dealing with the dilemma and finding reasonable solutions against the social isolation they could rely on an inter-professional team (with a strong *“team spirit”*), including volunteers and family members, and innovative technical solutions such as social media (34).

However, NDs often felt alone and exhausted by the responsibility. Similar results were reported by Behrens et al. who also found that the nursing home managers felt on their own and without support (35). Major factors contributing to these negative feelings were the perceived poor communication between NDs and health authorities, with a lack of clear guidance on how to deal with the dilemma, a sometimes difficult understanding of the priorities among the family members of residents, and the one-sided reporting style of mass media (36).

### Lessons learned and outlook

Asked for their wishes for the future in regard to the dilemma, NDs wished for more openly discussing the trade-off between (protective) regulations and autonomy / responsibility of nursing home residents. Ideally, this would also be mirrored by a more neutral reporting style of mass media. A better communication with employees, family members and authorities seemed crucial to the NDs to improve the coping with the dilemma. Finally some NDs wished for psychological support in order to prevent their exhaustion. The main findings of our study may inform stakeholders to integrate the support of NDs and their teams into their strategies and policies of coping with future pandemics. For example, the mass media and the public could develop a standard of commitment to neutral reporting and ethical discussion. Authorities could develop a clearer communication strategy with NDs for the condition of a pandemic, provide systematic psychological support and technical support for digital communication channels. The stakeholders of quality movements in favor of quality improvement may take up our findings for planning quality improvement activities. Finally, our results may inspire other research groups to further explore the dilemma in favor of the vulnerable group of nursing home residents.

As a lesson learned from these findings, best practices should be defined for a better preparedness of professionals, in order to meet the needs of the vulnerable population of nursing home residents (37).

### Strengths and limitations

There has been a remarkable research activity regarding nursing homes during the COVID-19 pandemic, including psychological and social issues (38–40). However, to the best of our knowledge, the perspective of NDs has not specifically been addressed in Switzerland yet. As a strength of our study, we filled this gap by not only exploring NDs’ perception of the dilemma between protective measures and isolation among residents, but also by investigating how they perceived their own situation and how they, and the entire nursing home team, were dealing with it, including factors hindering or helpful for their coping.

The participation rate of 38% seems a limitation regarding external validity. However, considering NDs’ high administrative workload, it is not so surprising. We tried to counterbalance this fact by addressing all of the NDs in German-speaking Switzerland.

## Conclusion

NDs are highly aware of the dilemma between protective measures and the burden of social isolation of nursing home residents during the COVID-19 pandemic and developed an impressive bundle of strategies for coping with it, together with other professionals, family members and authorities. As NDs are burdened themselves by the responsibility of how to deal best with this dilemma, they need support for a better preparedness in the future, including a better well-being of themselves. Our findings provide insights into what issues to consider for developing such supportive strategies.

### Key points

- The COVID-19 pandemic resulted in a number of regulatory protective measures for nursing homes, unintendedly leading to social isolation of their residents.
- Our cross-sectional mixed-method study explored how nursing home directors (NDs) perceived the implementation of protective measures and the social isolation of the residents, and how NDs were coping with the situation.
- NDs were highly aware of the dilemma between protection and avoiding social isolation, and developed a bundle of strategies to cope with.
- NDs reported to need more support, in favor of a better preparedness for future similar conditions.
- Our study findings may ultimately contribute to improve the care quality for nursing home residents and to reduce psychological stress of responsible professionals.

## Data availability statement

The original contributions presented in the study are included in the article/Supplementary material, further inquiries can be directed to the corresponding author.

## Ethics statement

Due to the nature of the study, no ethics approval was necessary and the local Ethics Committee of the Canton of Zurich confirmed that the study did not fall under the scope of the national Human Research Act (BASEC-Nr. Req-2022-00153). Participation in the survey was voluntary and all participants accepted a general informed consent informing about the purpose and aims of the study. All participants agreed to the publication of their anonymized data. The study was conducted in accordance with the Declaration of Helsinki (Association, 2014).

## Author contributions

SG: Conceptualization, Data curation, Formal analysis, Methodology, Project administration, Writing – original draft, Writing – review & editing. SD: Formal analysis, Writing – review & editing, Visualization. AP: Conceptualization, Data curation, Formal analysis, Methodology, Project administration, Writing – review & editing. OS: Conceptualization, Data curation, Formal analysis, Methodology, Project administration, Supervision, Writing – review & editing. SN-J: Conceptualization, Data curation, Formal analysis, Methodology, Project administration, Supervision, Writing – review & editing.
