# Supplementary material for: Perceived dilemma between protective measures and social isolation in nursing homes during the COVID-19 pandemic: a mixed methods study among Swiss nursing home directors
Source: Front Public Health. 2024 Mar 11;12:1292379. doi: 10.3389/fpubh.2024.1292379 (PMC10962325; doi:10.3389/fpubh.2024.1292379)
Supplement: Supplementary file 1 [file Data_Sheet_1.docx]

**
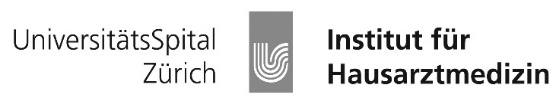

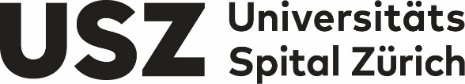
**

**Supplement 1: Invitation letter and content of the survey**

**COVID-19 pandemic**

**Survey on the Ethical Dilemma between Preventive Protective Measures and Social Isolation**

Dear Ladies and Gentlemen,

As you know very well from your own experience, nursing homes are particularly affected by the ongoing COVID-19 pandemic. Preventive protective measures help to reduce the risk of infection among residents who are particularly at risk. At the same time, however, there is a dilemma between the implementation of preventive protection measures and the consequences of social isolation.

We would like to investigate which preventive protective measures have been taken during the COVID-19 pandemic in retirement and nursing homes in German-speaking Switzerland. In addition, we would like to know about your observations to what extent individual preventive protective measures led to social isolation among residents, and to learn more about the burden of these measures on residents from your point of view.

Your answers will be treated confidentially, will not be forwarded, and will be evaluated anonymously. You can pause the survey at any time and continue it later.

We will publish the results of this survey. With your answers, together we can make a difference for retirement and nursing homes and their residents. As a small thank you for your participation, you have the opportunity to take part in a raffle of five gift baskets with a value of CHF 100 each (at the end of the survey).

Thank you very much for your valuable contribution, your opinion is important to us!

Your survey team

The following questions are to be answered by you regarding the dilemma between the individual preventive protective measures in relation to social isolation:

1. Has this protective measure been implemented at any time in your nursing home?
2. How has medical and non-medical care changed in your nursing home?
3. To what extent has the protective measure led to social isolation among the residents of the nursing home?
4. How much of a burden was this protective measure to place for the residents of the nursing home?

In the following questions, various preventive protective measures are presented. Please respond whether these protective measures have been implemented at any time in your nursing home.

In addition, we request your assessment of the extent to which these protective measures have led to social isolation among the residents and how high the burden (of your residents by these protective measures) was from your point of view.

IMPORTANT: Our questions refer to implemented preventive protective measures. They do not refer to special measures for residents who had direct contact with COVID-19 patients (quarantine) or measures for COVID-19 patients themselves (isolation).

* Gender-neutral language: For a better readability, only the masculine form is used in some of the texts. However, the formulations refer to members of all genders equally.

**Chapter 1: Physical Distancing (Part: 1/7)**

**Was this protective measure implemented at any time in your institution?**

- Distance rules among residents (e.g., distance 1.5 m at the dining table)

*Mandatory field

- Yes
- No
- Don’t know
- Different meal times for residents

*Mandatory field

- Yes
- No
- Don’t know
- Residents were allowed to use the common areas only under restriction of the number of people

*Mandatory field

- Yes
- No
- Don’t know
- Residents were not allowed to leave their rooms

*Mandatory field

- Yes
- No
- Don’t know
- Residents were not allowed to leave their department

*Mandatory field

- Yes
- No
- Don’t know
- Residents were not allowed to leave the entire nursing home

*Mandatory field

- Yes
- No
- Don’t know

**Please think about:**

**To what extent has the protective measure led to social isolation among nursing home residents?**

- Distance rules among residents (e.g., distance 1.5 m at the dining table)

*Mandatory field

- Not at all
- A little
- Moderate
- Strong
- Very strong
- NA
- Different meal times for residents

*Mandatory field

- Not at all
- A little
- Moderate
- Strong
- Very strong
- NA
- Residents were allowed to use the common areas only under restriction of the number of people

*Mandatory field

- Not at all
- A little
- Moderate
- Strong
- Very strong
- NA
- Residents were not allowed to leave their rooms

*Mandatory field

- Not at all
- A little
- Moderate
- Strong
- Very strong
- NA
- Residents were not allowed to leave their department

*Mandatory field

- Not at all
- A little
- Moderate
- Strong
- Very strong
- NA
- Residents were not allowed to leave the entire nursing home

*Mandatory field

- Not at all
- A little
- Moderate
- Strong
- Very strong
- NA

**Please think about:**

**How much of a burden was this social isolation for nursing home residents?**

- Distance rules among residents (e.g., distance 1.5 m at the dining table)

*Mandatory field

- Not burdensome all
- A little burdensome
- Moderately burdensome
- Strongly burdensome
- Very strongly burdensome
- NA
- Different meal times for residents

*Mandatory field

- Not burdensome at all
- A little burdensome
- Moderately burdensome
- Strongly burdensome
- Very strongly burdensome
- NA
- Residents were allowed to use the common areas only under restriction of the number of people

*Mandatory field

- Not burdensome at all
- A little burdensome
- Moderately burdensome
- Strongly burdensome
- Very strongly burdensome
- NA
- Residents were not allowed to leave their rooms

*Mandatory field

- Not burdensome at all
- A little burdensome
- Moderately burdensome
- Strongly burdensome
- Very strongly burdensome
- NA
- Residents were not allowed to leave their department

*Mandatory field

- Not burdensome at all
- A little burdensome
- Moderately burdensome
- Strongly burdensome
- Very strongly burdensome
- NA
- Residents were not allowed to leave the entire nursing home

*Mandatory field

- Not burdensome at all
- A little burdensome
- Moderately burdensome
- Strongly burdensome
- Very strongly burdensome
- NA

**Chapter 2: Visitors' restrictions (Part: 2/7)**

**Was this protective measure implemented at any time in your institution?**

- The number of visitors per visit was limited

*Mandatory field

- Yes
- No
- Don’t know
- The number of visitors per day/week was limited

*Mandatory field

- Yes
- No
- Don’t know
- Visits were allowed only by closest family members or friends

*Mandatory field

- Yes
- No
- Don’t know
- Visits were made in special visitor rooms

*Mandatory field

- Yes
- No
- Don’t know
- Visits made outside

*Mandatory field

- Yes
- No
- Don’t know
- Access for visitors according to current regulations "3G rule, 2G rule, 2G Plus rule"

*Mandatory field

- Yes
- No
- Don’t know
- Visitation restrictions (with exceptions, e.g., for emergency or palliative situations)

*Mandatory field

- Yes
- No
- Don’t know
- Absolute visitors' ban (without any exceptions)

*Mandatory field

- Yes
- No
- Don’t know

**Please think about:**

**To what extent has the protective measure led to social isolation among nursing home residents?**

- Distance rules among residents (e.g., distance 1.5 m at the dining table)

*Mandatory field

- Not at all
- A little
- Moderate
- Strong
- Very strong
- NA
- Different meal times for residents

*Mandatory field

- Not at all
- A little
- Moderate
- Strong
- Very strong
- NA
- Residents were allowed to use the common areas only under restriction of the number of people

*Mandatory field

- Not at all
- A little
- Moderate
- Strong
- Very strong
- NA
- Residents were not allowed to leave their rooms

*Mandatory field

- Not at all
- A little
- Moderate
- Strong
- Very strong
- NA
- Residents were not allowed to leave their department

*Mandatory field

- Not at all
- A little
- Moderate
- Strong
- Very strong
- NA
- Residents were not allowed to leave the whole nursing home

*Mandatory field

- Not at all
- A little
- Moderate
- Strong
- Very strong
- NA

**Please think about:**

**How much of a burden was this social isolation for nursing home residents?**

- Distance rules among residents (e.g., distance 1.5 m at the dining table)

*Mandatory field

- Not burdensome at all
- A little burdensome
- Moderately burdensome
- Strongly burdensome
- Very strongly burdensome
- NA
- Different meal times for residents

*Mandatory field

- Not burdensome at all
- A little burdensome
- Moderately burdensome
- Strongly burdensome
- Very strongly burdensome
- NA
- Residents were allowed to use the common areas only under restriction of the number of people

*Mandatory field

- Not burdensome at all
- A little burdensome
- Moderately burdensome
- Strongly burdensome
- Very strongly burdensome
- NA
- Residents were not allowed to leave their rooms

*Mandatory field

- Not burdensome at all
- A little burdensome
- Moderately burdensome
- Strongly burdensome
- Very strongly burdensome
- NA
- Residents were not allowed to leave their department

*Mandatory field

- Not burdensome at all
- A little burdensome
- Moderately burdensome
- Strongly burdensome
- Very strongly burdensome
- NA
- Residents were not allowed to leave the whole nursing home

*Mandatory field

- Not burdensome at all
- A little burdensome
- Moderately burdensome
- Strongly burdensome
- Very strongly burdensome
- NA

**Chapter 3: Physician and non-physician care contacts (Part: 3/7)**

***Compare to the supply situation before the pandemic began when answering the following questions.***

How is medical care organized at your institution?

- Physicians employed by the nursing home
- GP-based system
- Mixed system
- Others

**Please think about:**

**How has physician and non-physician care changed at your nursing home?**

***Phyisicians’ care***

- Physician consultations in the practice

*Mandatory field

- No change
- Reduced
- Not provided at all
- Don’t know
- Visits by the general practitioner or the nursing home physician

*Mandatory field

- No change
- Reduced
- Not provided at all
- Don’t know

***Therapeutic care***

- Physiotherapy services

*Mandatory field

- No change
- Reduced
- Not provided at all
- Don’t know
- Logopedic services

*Mandatory field

- No change
- Reduced
- Not provided at all
- Don’t know

***Nursing care***

- Carrying out personal hygiene or body-related measures

*Mandatory field

- No change
- Reduced
- Not provided at all
- Don’t know
- Assistance to improve mobility (mobilization to chair, gait training, etc.)

*Mandatory field

- No change
- Reduced
- Not provided at all
- Don’t know
- Carrying out of psychological conversations on the part of the nursing staff

*Mandatory field

- No change
- Reduced
- Not provided at all
- Don’t know

***Spiritual care***

- Visits by the pastoral counselor (e.g., religious conversations or church services)

*Mandatory field

- No change
- Reduced
- Not provided at all
- Don’t know
- Spiritual care for the dying

*Mandatory field

- No change
- Reduced
- Not provided at all
- Don’t know

**Please think about:**

**To what extent has the protective measure led to social isolation among nursing home residents?**

***Phyisicians’ care***

- Physician consultations in the practice

*Mandatory field

- Not at all
- A little
- Moderate
- Strong
- Very strong
- NA
- Visits by the general practitioner or the nursing home physician

*Mandatory field

- Not at all
- A little
- Moderate
- Strong
- Very strong
- NA

***Therapeutic care***

- Physiotherapy services

*Mandatory field

- Not at all
- A little
- Moderate
- Strong
- Very strong
- NA
- Logopedic services

*Mandatory field

- Not at all
- A little
- Moderate
- Strong
- Very strong
- NA

***Nursing care***

- Carrying out personal hygiene or body-related measures

*Mandatory field

- Not at all
- A little
- Moderate
- Strong
- Very strong
- NA
- Assistance to improve mobility (mobilization to chair, gait training, etc.)

*Mandatory field

- Not at all
- A little
- Moderate
- Strong
- Very strong
- NA
- Carrying out of psychological conversations on the part of the nursing staff

*Mandatory field

- Not at all
- A little
- Moderate
- Strong
- Very strong
- NA

***Spiritual care***

- Visits by the pastoral counselor (e.g. religious conversations or church services)

*Mandatory field

- Not at all
- A little
- Moderate
- Strong
- Very strong
- NA
- Spiritual care for the dying

*Mandatory field

- Not at all
- A little
- Moderate
- Strong
- Very strong
- NA

**Please think about:**

**How much of a burden was this measure for nursing home residents?**

***Phyisicians’ care***

- Physician consultations in the practice

*Mandatory field

- Not burdensome at all
- A little burdensome
- Moderately burdensome
- Strongly burdensome
- Very strongly burdensome
- NA
- Visits by the general practitioner or the nursing home physician

*Mandatory field

- Not burdensome at all
- A little burdensome
- Moderately burdensome
- Strongly burdensome
- Very strongly burdensome
- NA

***Therapeutic care***

- Physiotherapy services

*Mandatory field

- Not burdensome at all
- A little burdensome
- Moderately burdensome
- Strongly burdensome
- Very strongly burdensome
- NA
- Logopedic services

*Mandatory field

- Not burdensome at all
- A little burdensome
- Moderately burdensome
- Strongly burdensome
- Very strongly burdensome
- NA

***Nursing care***

- Carrying out personal hygiene or body-related measures

*Mandatory field

- Not burdensome at all
- A little burdensome
- Moderately burdensome
- Strongly burdensome
- Very strongly burdensome
- NA
- Assistance to improve mobility (mobilization to chair, gait training, etc.)

*Mandatory field

- Not burdensome at all
- A little burdensome
- Moderately burdensome
- Strongly burdensome
- Very strongly burdensome
- NA
- Carrying out of psychological conversations on the part of the nursing staff

*Mandatory field

- Not burdensome at all
- A little burdensome
- Moderately burdensome
- Strongly burdensome
- Very strongly burdensome
- NA

***Spiritual care***

- Visits by the pastoral counselor (e.g., religious conversations or church services)

*Mandatory field

- Not burdensome at all
- A little burdensome
- Moderately burdensome
- Strongly burdensome
- Very strongly burdensome
- NA
- Spiritual care for the dying

*Mandatory field

- Not burdensome at all
- A little burdensome
- Moderately burdensome
- Strongly burdensome
- Very strongly burdensome
- NA

***Physician and non-physician care contacts.***

***Summary questions:***

**In your personal perception: Do you think that physician and non-physician care was increasingly provided by telephone or video communication?**

*Mandatory field

- Yes
- No
- Don’t know

*If the answer is –yes- , please mark the answers that apply (multiple answers possible):*

- Medical care
- Therapeutic care
- Spiritual care

**In your personal perception: Do you think the COVID-19 pandemic had a negative impact on the quality of physician care at your nursing home?**

*Mandatory field

- I cannot evaluate
- No negative impact at all
- Moderately negative impact
- Strongly negative impact
- Very strongly negative impact

*If you had perceived negative effects on the quality of physician care, what reasons do you suspect for this:*

**In your personal perception: Do you think the COVID-19 pandemic had a negative impact on the quality of non-physician care at your nursing home?**

*Mandatory field

- I cannot evaluate
- No negative impact at all
- Moderately negative impact
- Strongly negative impact
- Very strongly negative impact

*If you perceived negative effects on the quality of non-physician care, what reasons do you suspect for this?*

**Chapter 4: Group and community opportunities (Part: 4/7)**

**Please think about:**

**How have group and community services changed at your nursing home?**

- In relation to frequency of offerings: Group offerings/community offerings (e.g., activation therapy, group gymnastics, singing, handwork, cooking, and home concerts).

*Mandatory field

- No change
- Reduced
- Not provided at all
- Don’t know
- In relation to the number of participants: Group offers/community offers (e.g., activation therapy, group gymnastics, singing, handicrafts, cooking, and home concerts).

*Mandatory field

- No change
- Reduced
- Not provided at all
- Don’t know

**Please think about:**

**To what extent has the protective measure led to social isolation among nursing home residents?**

- In relation to frequency of offerings: Group offerings/community offerings (e.g., activation therapy, group gymnastics, singing, handwork, cooking, home concerts).

*Mandatory field

- Not at all
- A little
- Moderate
- Strong
- Very strong
- NA
- In relation to the number of participants: group offers/community offers (e.g. activation therapy, group gymnastics, singing, handicrafts, cooking, house concerts).

*Mandatory field

- Not at all
- A little
- Moderate
- Strong
- Very strong
- NA

**Please think about:**

**How much of a burden was this social isolation for nursing home residents?**

- In relation to frequency of offerings: Group offerings/community offerings (e.g., activation therapy, group gymnastics, singing, handwork, cooking, home concerts).

*Mandatory field

- Not burdensome at all
- A little burdensome
- Moderately burdensome
- Strongly burdensome
- Very strongly burdensome
- NA
- In relation to the number of participants: group offers/community offers (e.g. activation therapy, group gymnastics, singing, handicrafts, cooking, house concerts).

*Mandatory field

- Not burdensome at all
- A little burdensome
- Moderately burdensome
- Strongly burdensome
- Very strongly burdensome
- NA

**Chapter 5: Handling the dilemma (Part: 5/7)**

- **What measures have you taken in-house to mitigate the dilemma?**

*Mandatory field

- **Who or what supported you in overcoming the dilemma?**

*Mandatory field

- **Who or what has hindered you in dealing with the dilemma?**

*Mandatory field

- **What would you do differently in future pandemic phases?**

*Mandatory field

**Chapter 6: Personal details of the COVID-19 pandemic (Part: 6/7)**

- How much of a burden did you personally feel the COVID-19 pandemic placed on you while doing your job? I felt…
- Very strongly burdened
- Strongly burdened
- Moderately burdened
- Slightly burdened
- Not burdened at all
- Were there any positive effects of the COVID 19 pandemic? If yes, what effects?
- What support would you wish for to cope with future pandemics? From whom should they come?
- What other observations related to the dilemma have you made?

**Chapter 7: Information about the participant and the institution (Part: 7/7)**

- What is your gender?
  - Female
  - Male
  - Divers
- How old are you?
- What is your position in the nursing home?
- Nursing Home Manager
- Head of Nursing Service
- Ward Manager
- Nursing Home CEO
- Quality Manager
- Others
- For how many years have you been working in this position (work experience)?
- < 5 years
- 5 – 15 years
- > 15 years
- In which canton is, your nursing home located? Please write the respective canton in the text box.
- Location of the institution
- Urban
- Suburb
- Rural
- How many inpatient care places (long-term places) are there at your nursing home?

*Please write the number of inpatient care places in the text box.*

- On average, how long do residents live in your nursing home?

*Please indicate a length of time in years and months.*
