# Supplementary material for: Perceived dilemma between protective measures and social isolation in nursing homes during the COVID-19 pandemic: a mixed methods study among Swiss nursing home directors
Source: Front Public Health. 2024 Mar 11;12:1292379. doi: 10.3389/fpubh.2024.1292379 (PMC10962325; doi:10.3389/fpubh.2024.1292379)
Supplement: Supplementary file 4 [file Data_Sheet_4.docx]

**Supplement 4. Baseline characteristics of the study participants and the participating institutions**

| Participants |  |
| --- | --- |
| Sex (*n* = 290) |  |
| Female | 182 (62.8%) |
| Age, years, median [IQR] (*n* = 290) | 55 [48-58] |
| Position (*n* = 291) |  |
| Nursing Home Manager | 155 (53.3%) |
| Head of Nursing Service | 82 (28.2%) |
| Ward Manager | 9 (3.1%) |
| Nursing Home CEO | 16 (5.5%) |
| Quality Manager | 11 (3.8%) |
| Others | 18 (6.2%) |
| Working experience in the current position (*n* = 291) |  |
| < 5 years | 88 (30.2%) |
| 5 – 15 years | 127 (43.6%) |
| > 15 years | 76 (26.1%) |
| Institutions | |
| Location (*n* = 288) |  |
| Urban | 27 (9.4%) |
| Suburb | 93 (32.3%) |
| Rural | 168 (58.3%) |
| Number of beds (*n* = 286) |  |
| < 50 | 83 (29.0%) |
| 50 - 100 | 127 (44.4%) |
| 101 - 200 | 63 (22.0%) |
| 201 - 300 | 12 (4.2%) |
| > 301 | 1 (0.4%) |
| Average duration of residents’ stay (*n* = 257) |  |
| < 12 months | 5 (2.0%) |
| 1 – 2 years | 70 (27.2%) |
| >2 – 5 years | 157 (61.1%) |
| >5 - 10 years | 18 (7.0%) |
| > 10 years | 5 (2.0%) |
| > 20 years | 2 (0.8%) |
| Organization of physicians’ care for residents (*n* = 342) |  |
| Physicians employed by the nursing home | 67 (19.6%) |
| GP-based system | 132 (38.6%) |
| Mixed system | 138 (40.3%) |
| Others | 5 (1.5%) |

*IQR = Interquartile Range; GP = General Practitioner
