# Supplementary material for: Perceived dilemma between protective measures and social isolation in nursing homes during the COVID-19 pandemic: a mixed methods study among Swiss nursing home directors
Source: Front Public Health. 2024 Mar 11;12:1292379. doi: 10.3389/fpubh.2024.1292379 (PMC10962325; doi:10.3389/fpubh.2024.1292379)
Supplement: Supplementary file 5 [file Data_Sheet_5.docx]

| **Dimension** pre-defined by the open-text question  **Supplement 5. Overview of dimensions, themes, subthemes and corresponding citations (examples)** | **Themes** | **Subthemes** | **Citations (examples)** |
| --- | --- | --- | --- |
| Quality of medical service | Physical contact with physician | - Self-protection from infection - Accessibility - Medical prioritization | *“Physicians were afraid to get infected themselves.”*  *“Physicians are visiting patients in severe cases only.”* |
|  | Overload | - Lack of time - High workload | *“General practitioners were overwhelmed by the situation and had fewer resources for the nursing homes.”*  *“The lack of professionals, such as physicians, pharmacists, physiotherapists, etc. is fundamentally a problem.”* |
|  | Interprofessional collaboration | - Collaboration with external providers - In-house collaboration - Inappropriate delegation of tasks | *“Support from specialists/consultants more difficult.”*  *“Teamwork between physicians and nursing staff has suffered greatly.”*  *“Increased transfer of activities from doctors to nursing care/institution.”* |
| Quality of non-medical service | Overload | - Lack of time - High workload   and shortage of healthcare staff | *“Organisational issues, communication, documentation and protective measures required a lot of time, so there was less time for the care of residents.”* |
|  | Reduction of non-nursing care services | - Physiotherapy - Activation therapy - Spiritual care | *“Physiotherapy was no longer possible - some of the residents have significantly lost their fitness/mobility.”*  *"Activities were delayed or cancelled, which reduced the quality.”*  *"The residents were missing church services."* |
| In-house measures to cope with the dilemma | Innovative offers of activation | - Activation in smaller groups - New options of activation | *“There were more individual visits by spiritual carers, skype for residents and next of kin, setup of a visitors’ room.”*  *“Concerts to join at the balcony, a visitors’ tent, tablets for using zoom, volunteers for visiting residents individually, taking a walk with them, having a phone or skype call with them, writing a letter to them. School classes were asked for support by writing letters or giving a drawing as a present.”* |
|  | Internal and external communication | - | *“Much internal and external communication with residents, family members and staff.”* |
|  | Liberal implementation of protection measures | - | *“We have tried to find the optimum balance between 'living' and measures in consultation with residents and family members.”* |
|  | Recruitment of personnel | - Increase in skilled personnel - Use of non-specialist personnel | *“Every employee tried to substitute a part of the lacking offers.”* |
| Support for coping with the dilemma | Institutions and associations | - | *"An additional person was employed for activation during the complete lockdown, each employee tried to make up for some of the missing services (hairdresser, physio), as many individual appointments as possible."* |
|  | Team | - Employees - Volunteers - Spiritual carers - General practitioner or geriatrician responsible for the nursing home - Family members - Other nursing home residents | *“There was a strong team spirit and the confidence to manage, together in the multidisciplinary team”*  *"...good cooperation with ... pastor."*  *"Interdisciplinary meetings, with the Federal Authority of Public Health, the cantonal responsibles for health, the general practitioners and the doctors in the area.”*  *"Informing residents about protective measures - conveying a sense of purpose."* |
|  | No external support | - | *"Nobody (supported me), (I had to rely on) own resources if available."* |
|  | Technical innovation | - | *"Skype"*  *"Video messages"* |
|  | No dilemma perceived | - | *"What dilemma???"* |
| Barriers towards coping with the dilemma | Public authorities and institutions | - Inadequate regulation | *“Changing and confusing regulations by the authorities.”*  *"... the rapid changes could not always be implemented promptly. Federal and cantonal requirements, related cantonal regulations (were barriers)."* |
|  | Family members | - | *“A poor understanding (of measures) among family members.”* |
|  | Media | - | *"One-sided reporting in the media."* |
|  | Lack of resources | - | *"The constant loss of staff."*  *"Limited space and resources."* |
|  | Uncertainty about pandemic progression | - | *“Not knowing how long and how intensely the pandemic will develop.”* |
|  | No barrier | - | *“Nothing or nobody (was a barrier).”* |
| Lessons learned | Adapt concepts | - Prevent isolation - Stock material - Involve residents in decision making - Plan escalation step - Increase ratio of personnel | *"Weighing up what is more important – end-of-life quality of life or end-of-life prolongation of life under isolating, saddening measures."*  *"Visiting bans should be reviewed again."*  *"Giving residents more personal responsibility. Accept their decision."*  *"We have now described escalation levels in the concept of communicable diseases. The concept is transferable to all possible infectious diseases. This means we are ready to act from day one and everyone knows what measures need to be taken."*  *"Immediate increase in nursing staff (yes, this is probably an illusion) As much as necessary, as little as possible."* |
|  | More autonomy for residents | - | *“I would hand over more responsibility to the residents and accept their decisions.”* |
|  | Improve communication | - | *“To better communicate with employees, family members (using e-mail) and to better collaborate with authorities.*  *“To establish regular consultations for family members.””* |
|  | No adaption | - | *“No adaption (necessary).”* |
| Potentially positive impact of the pandemic | Better preparedness for a next pandemic | - | *“The next pandemic can come; we are ready and equipped with know-how.”* |
|  | Thankfulness of the family members | - | *“Thankfulness of residents and their relatives - understanding for measures taken - spontaneous gifts and praise for our work.”* |
|  | Team spirit  Solidarity and collaboration between departments | - Team - Residents | *“Our team has grown together very strongly; in a positive sense, we are at a completely different level than before the pandemic.”*  *“Cohesion among residents has increased a lot.”* |
|  | Self-confidence | *-* | *“We have grown with the challenges.”* |
|  | More quiet atmosphere | - | *“People suffering from dementia had more quiet and were therefore more balanced and satisfied.”* |
|  | Focus on the relevant things | - | *“Yes, setting priorities; we have put the resident's wishes even more in the centre.”*  *“Focus on the essentials.”* |
|  | Digital communication | - Residents - Nursing home directors | *“Information for family members could be set up electronically.”*  *“Working from home has become more important.”* |
|  | Hygiene issues | - Increase of knowledge - Implementation - Consequences | *“Dealing with hygiene has definitely improved.”*  *“There were hardly any short-term absences during the first phase. Fewer other 'flu cases' throughout the phases.”* |
| Wishes for support for future pandemics | Mass Media | - | *“More objective reporting by the media. Only the negative aspects were covered.”* |
|  | Communication with third parties | - | *“Clearer and more timely communication about the upcoming measures from the responsible authorities.”* |
|  | Resources | - Human resources - Material resources | *“More hands and hearts that could be used immediately when needed - that would be nice.”* |
|  | Autonomy | - | *“Less regulations and more responsibility for the nursing homes and the residents, in accordance with the family members.”* |
|  | Psychological support | - | *“Support at the upper management level too, not just for employees - the management also has a soul.”* |
|  | End-of-life care | - | *“The topic of dying must be shifted to the society. It has to be possible to openly discuss this with residents and family members”* |
| Further free comments related to the dilemma | Reaction to the pandemic | - Residents - Nursing home directors - Family members - Nursing team | *“There was a huge burden for the care team, physically and psychologically; with no support measures. Many of them quit their job after the pandemic.”* |
|  | Importance of family members | - | *“The dilemma between protection and autonomy of residents brought me to my limits, and I am still tired and exhausted. The responsibility was enormous.”* |
|  | Egoism | - | *“Sometimes I get the impression that people have become even) more selfish.”* |
|  | Calmness and slowing down | - | *“On the whole, a great deal of serenity was observed among the older generation.”*  *“Increasing serenity among employees.”* |
| Residents with dementia and cognitive impairment | Communication and understanding | - | *“Wearing face masks hindered communication and caring substantially, especially for people with cognitive impairment.”* |
|  | Untargeted stimuli | - | *“There was less agitation on dementia wards due to less stimuli from outside.”* |
|  | Perception of protective measures | - | *“The residents were very relaxed. They did not even perceive the restrictions”* |
|  | Exceptions from rules and regulations | - | *“The regional authority provided the option that they were allowed having exceptional regulations for people with dementia.”* |
